# Robot-Assisted Laparoscopic IVC Treatment Strategy in Retroperitoneal Tumors

**DOI:** 10.3389/fonc.2022.908272

**Published:** 2022-05-20

**Authors:** Lei Liu, Shiying Tang, Zhuo Liu, Cheng Liu, Hongxian Zhang, Xiaojun Tian, Guoliang Wang, Shudong Zhang, Lulin Ma

**Affiliations:** Department of Urology, Peking University Third Hospital, Haidian District, Beijing, China

**Keywords:** robot-assisted, inferior vena cava (IVC), treatment strategy, retroperitoneal tumors, surgical technique

## Abstract

**Objectives:**

To show the practice of robot-assisted laparoscopic inferior vena cava (IVC) treatment strategies in patients with retroperitoneal tumors.

**Patients and Methods:**

From October 2020 to July 2021, 17 patients with retroperitoneal tumors successfully underwent robot-assisted laparoscopic tumor resection with IVC management. The patient details, tumor characteristics, intraoperative data, pathological features and severe complications were assessed. The IVC treatment strategies were divided into 4 ways: ①local resection and primary repair of the IVC; ②IVC ligation; ③ IVC reconstruction by bovine pericardial grafts; and ④ IVC transection and anastomosis.

**Results:**

In terms of IVC management, 5 cases had conventional total occlusion of the IVC and its branches, 3 cases had delayed occlusion of the proximal IVC technique, 2 cases had IVC resection by Satinsky clamp, 5 cases had IVC ligation, 1 case had IVC reconstruction by bovine pericardial grafts and 1 case had IVC transection and anastomosis. The median operation time was 151 min, and blood loss was 500 ml. There was no severe complication perioperatively. The follow-up time of 17 patients was 8 to 17 months (median: 12 months). No local recurrence or overall death was found during follow-up.

**Conclusions:**

These robot-assisted laparoscopic IVC treatment strategies were considered to be safe and feasible in experienced centers, as well as helpful to completely remove the tumor for better oncological prognosis and restore the blood reflux of IVC as much as possible to ensure fewer postoperative complications.

## Introduction

Retroperitoneal tumors frequently extend to the inferior vena cava (IVC) in advanced cases, including renal cell carcinoma (RCC), adrenal tumors, leiomyosarcoma, paraganglioma, etc. The tumors could invade the IVC wall directly or extend intravascularly to the IVC, resulting in the formation of IVC tumor thrombus (TT). Regardless of the histology and pattern of tumor invasion, surgical intervention should be performed for en bloc resection of the tumor and invaded IVC, and it seems to be the only treatment with the potential for cure (1). Neoadjuvant therapy is currently under investigation in RCC clinical trials. For metastatic RCC, cytoreductive nephrectomy combined with preoperative tyrosine kinase inhibitors (TKIs) could still show a survival benefit and the TKIs could also be used as adjuvant therapy after surgery (2). Although radical surgery can improve the long-term survival of patients with poor prognosis, simultaneous retroperitoneal tumor resection and IVC resection are associated with high surgical risk of bleeding and thromboembolism (3). IVC treatment strategies hold important status in the surgical procedure of retroperitoneal tumors.

IVC resection and reconstruction have been described in several previous studies and proved to be safe and effective in open surgery (4). Caitlin et al. described surgical procedures for primary repair, patch repair and graft reconstruction of IVCs, concluding that IVC resection and reconstruction for malignant tumors can be performed safely with low venous thromboembolic (VTE) mortality (4). In addition, previous studies also reported the use of bovine pericardium for IVC or hepatic portal vein reconstruction (5). The technique of IVC reconstruction provides a opportunity for complete malignant tumor resection to obtain preferable oncological outcomes for patients (6).

Minimally invasive technology can accelerate the recovery of patients, but it also increases the difficulty of surgery, especially in robot-assisted laparoscopic surgery. In addition, these surgical procedures seem to be complicated and require a sufficient preoperative surgical design. However, there are few studies and no formal guidelines on the systematic review of robot-assisted laparoscopic IVC treatment strategies in retroperitoneal tumors. Therefore, through the retrospective analysis of the operation procedures of 17 patients, the current study aimed to summarize and describe 6 different treatment strategies of IVC in a robot-assisted laparoscopic approach.

## Patients and Methods

### Patients

We retrospectively analyzed the clinicopathological data of 17 patients with retroperitoneal tumors in our hospital from October 2020 to July 2021. All patients successfully underwent robot-assisted laparoscopic tumor resection with IVC management. Patient details (sex, age, clinical symptoms), tumor characteristics (tumor location, tumor size, Mayo Clinic classification, presence of venous thrombus preoperatively), intraoperative data (IVC management, operation time, blood loss), pathological features (histology, venous invasion in histology, tumor grade), severe complications, distant metastasis and adjuvant therapy were assessed.

All patients underwent enhanced computed tomography urography (CTU) and enhanced magnetic resonance imaging (MRI) examination of the IVC preoperatively to evaluate the side, size and location of the tumor, the length and maximum width of the TT (if it existed), and surrounding structures. Chest X-ray or lung CT examination was used to evaluate the presence of lung metastasis. Optional PET-CT was used to evaluate systemic metastasis. All patients completed B-ultrasound examination of lower extremity deep venous thrombosis before operation. Fifteen cases had retroperitoneal tumors on the right side and two on the left. IVCTT was classified according to the Mayo classification. Renal tumors were classified according to the 2017 eighth edition AJCC cancer staging manual (7). In terms of complications, the Clavien grading system was used, and grade III or IV refers to severe complications (8).

The study was approved by the ethics committee of the Peking University Third Hospital. All the patients signed written consent to allow the use of their data. All procedures were performed by a single chief physician of urology (Lulin Ma) with experience in robot-assisted laparoscopic surgery and vascular surgery, which is from the long-term clinical basis of renal transplantation.

### Surgical Procedures

According to the different surgical procedures of IVC, we divided the IVC treatment strategies into 4 ways: ① local resection and primary repair of the IVC; ② IVC ligation; ③ IVC reconstruction by bovine pericardial grafts; and ④ IVC transection and anastomosis. The first category ‘local resection and primary repair of the IVC’ can also be divided into ①conventional total occlusion of the IVC and its branches; ② delayed occlusion of the proximal IVC (DOPI) technique; and ③IVC resection by Satinsky clamp.

For right retroperitoneal tumors, after general anesthesia and Foley catheter placement, the patients were placed in the left lateral decubitus position with a chest pillow and tilted to the back by 30°. A Veress needle was inserted around the umbilicus to establish a pneumoperitoneum. A 12-mm trocar was inserted into the right boundary of the rectus muscle below the 11th rib, where the laparoscopic camera was placed. In direct view, two 8-mm robotic ports were separately placed on the right edge of the rectus muscle below the rib margin and the medial iliac spine near the rectus muscle. Then, two 12-mm trocars were separately placed on the anterior midline around the umbilicus and 8 cm above the umbilicus.

For left retroperitoneal tumors, the patient was placed in the right lateral decubitus position, and the placement of trocars was similar to that of the right retroperitoneal tumors. Routine steps were followed to isolate retroperitoneal tumors, IVCs and nearby arteries and veins, such as renal arteries and veins and reproductive vessels. According to different intraoperative conditions, different IVC treatment strategies were performed and are shown as follows:

#### Conventional Total Occlusion of IVC and Its Branches

Take the right retroperitoneal tumors as an example. The exposure of IVCs should be differentiated according to the different lengths of tumor thrombi invading the IVC. If necessary, several short hepatic veins should be ligated to obtain IVCs long enough for blocking. The right renal artery was easier to find and blocked between the IVC and the aorta. The right renal vein, left renal vein and the distal and proximal ends of the IVC were fully exposed and twined by double circles of vascular blocking band (eg. silastic vessi-loops). Then, the distal end of the IVC, left renal vein and proximal IVC were blocked in the proper sequence. The IVC wall was cut by scissors along the long axis of the IVC on the junction of the renal vein and IVC. The tumor and invaded IVC wall were completely removed by scissors and sutured with 4-0 Prolene suture continuously after rinsing with heparin saline to make the IVC full and avoid air embolism. The block of the proximal end of IVC, left renal vein, and the proximal end of IVC was removed in turn. It was necessary to achieve meticulous hemostasis, and surgical drains were left before routinely closing the incision in layers.

#### DOPI Technique

The front steps of the DOPI technique were similar to the conventional total occlusion of the IVC and its branches mentioned above. The significant difference was that the proximal end of the IVC was not blocked at once but still needed to be free. After blocking the uninjured renal vein and distal end of the IVC, the pneumoperitoneum pressure was raised to 15-25 cmH2O to reduce the blood flow from the proximal end of the IVC instead of blocking it. After removing the TT in the IVC, the lumen of the proximal end of the IVC became larger, and the blood reflux increased. Pneumoperitoneum pressure alone may not be enough to block the proximal flow of the IVC, while a bulldog clamp was needed to be placed at the proximal end of the IVC. This surgical technique was fully described in our previous study (9).

#### IVC Resection by Satinsky Clamp

Satinsky clamp could be used in RCC with Mayo level 0 or 1 TT, which existed in the renal vein or just entered the IVC. The renal vein was isolated fully until the entrance to the IVC and clipped with a Satinsky clamp, resulting in partial occlusion of the IVC. Then, the invaded renal vein and tumor thrombus were removed and sutured with 4-0 Prolene sutures after flushing with heparinized saline. The block was released, and it was observed whether there was obvious bleeding in the operative field.

#### IVC Ligation

**Figure 1:**
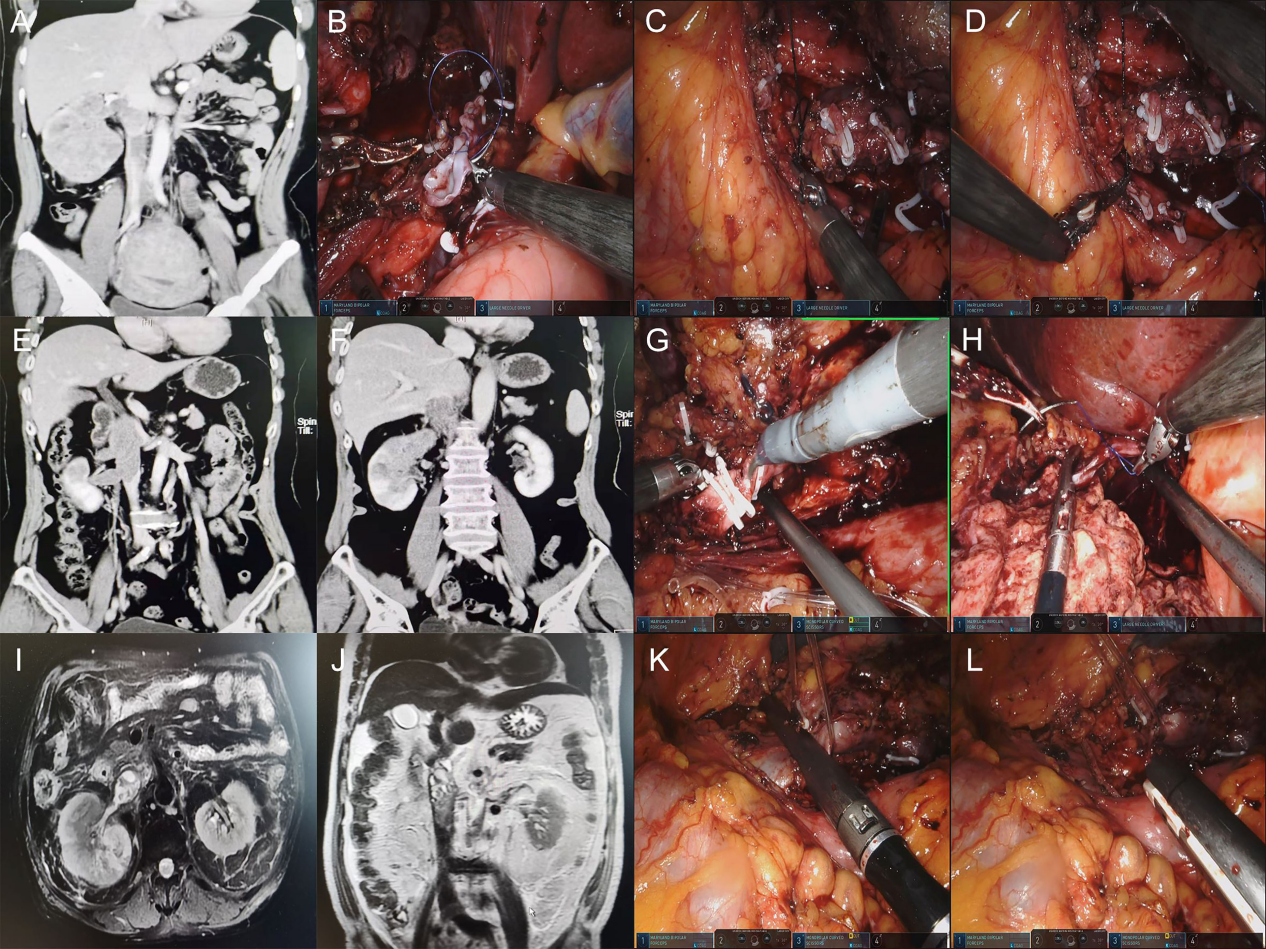
**(A, E, F, I, J)** Preoperative CTU images of retroperitoneal tumors with IVC tumor thrombus. **(B)** Suture the proximal IVC with a 4-0 Prolene vascular line. **(C, D)** Ligation of the distal end of IVC due to the thrombus below the tumor thrombus. **(G)** Ligation of the distal end of IVC by using double Hem-o-lock. **(H)** Proximal IVC ligation using a single Hem-o-lock and suture. **(K, L)** IVC Ligation by using Endo-GIA.

All cases with IVC ligation were right retroperitoneal tumors. Four cases were found in which the IVC was filled and blocked by TT. There was an organized thrombus below the TT, and the boundary between them was unclear. Another case showed adhesion between the TT and circumferential IVC wall. The distal end of the IVC and the left renal vein were blocked in turn, but the proximal end of the IVC did not need to be blocked. The IVC wall was cut by scissors along the entrance of the right renal vein into the IVC. The TT invaded the IVC wall tightly, and there was an organized thrombus with an unclear boundary below the TT. The invaded IVC was cut off, and the bilateral end of the IVC was closed by Endo-GIA or continuously sutured with a 4-0 Prolene vascular line after washing the lumen with heparin water. Then, the left renal vein and distal end of the IVC were unblocked in turn (**Figure 1**).

#### IVC Reconstruction by Bovine Pericardial Grafts

**Figure 2:**
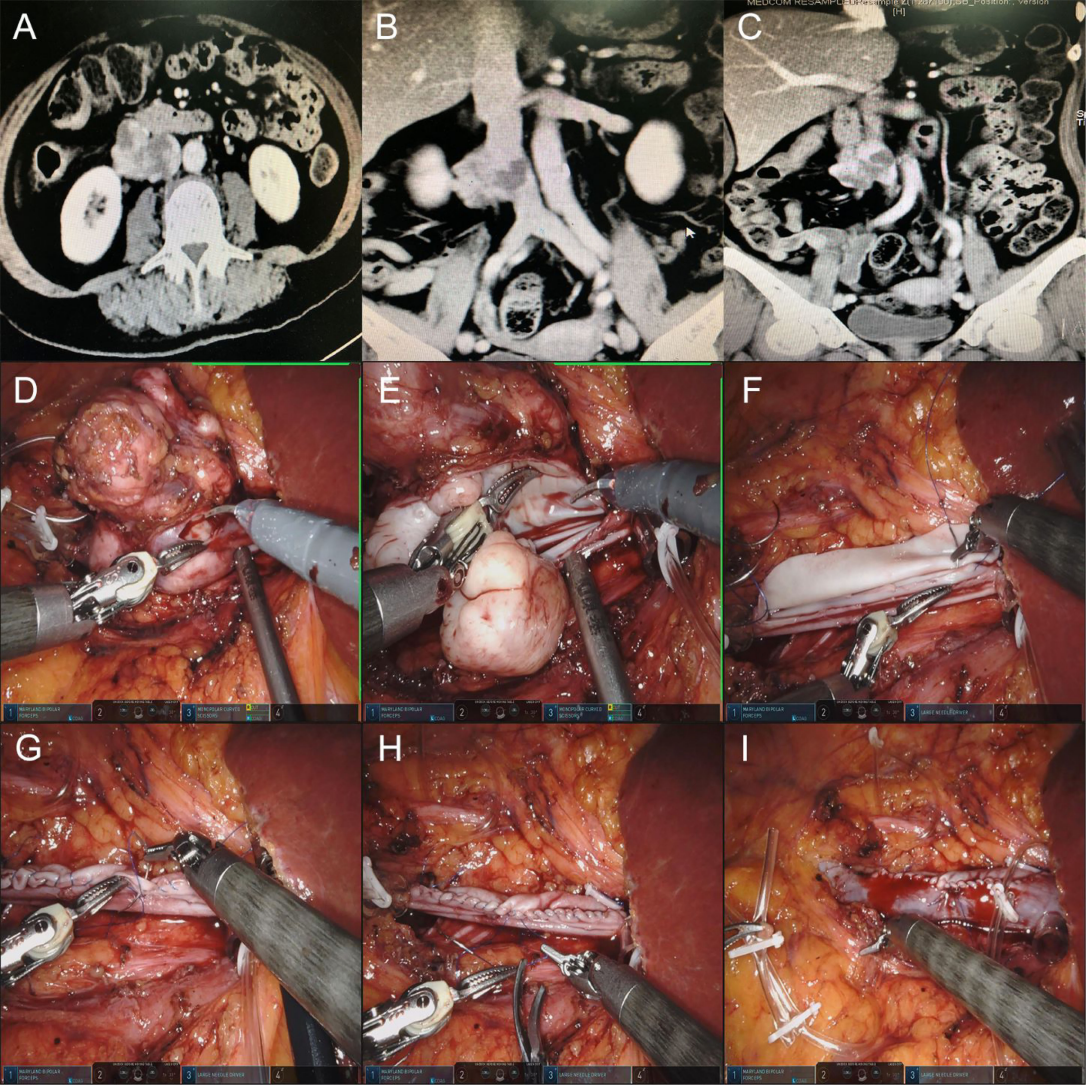
**(A–C)** Preoperative CTU images of the retroperitoneal tumor of patient 4. **(D, E)** Resection of retroperitoneal tumor and IVC tumor thrombus. **(F)** Suture the posterior wall of bovine pericardium graft and IVC wall continuously first. **(G, H)** Suture the anterior wall of bovine pericardium graft and IVC wall continuously. **(I)** Release the blocking of the IVC, and the IVC blood flow was unobstructed.

The pathology of this case was IVC leiomyosarcoma. It was difficult to separate the tumor from the IVC, which invaded approximately 5 cm. The decision to use bovine pericardial grafts was driven by the original biological structure of the IVC to reduce the occurrence of postoperative lower limb edema to the greatest extent. On the other hand, the IVC invaded by the tumor did not cross either side of the renal vein. Therefore, there was no need to reconstruct the renal vein after partial resection of the IVC. During the operation, the proximal and distal ends of the IVC were fully isolated and blocked. The tumor and partial IVC wall were completely resected. The area of the excised IVC was estimated, and the bovine pericardial graft was clipped to the appropriate size *in vitro*. The graft was sutured to the native IVC with a 4-0 Prolene vascular line continuous at either end. Before the suture was completed, the IVC was flushed with heparinized saline prior to removing clamps (**Figure 2**).

### IVC transection and anastomosis

**Figure 3:**
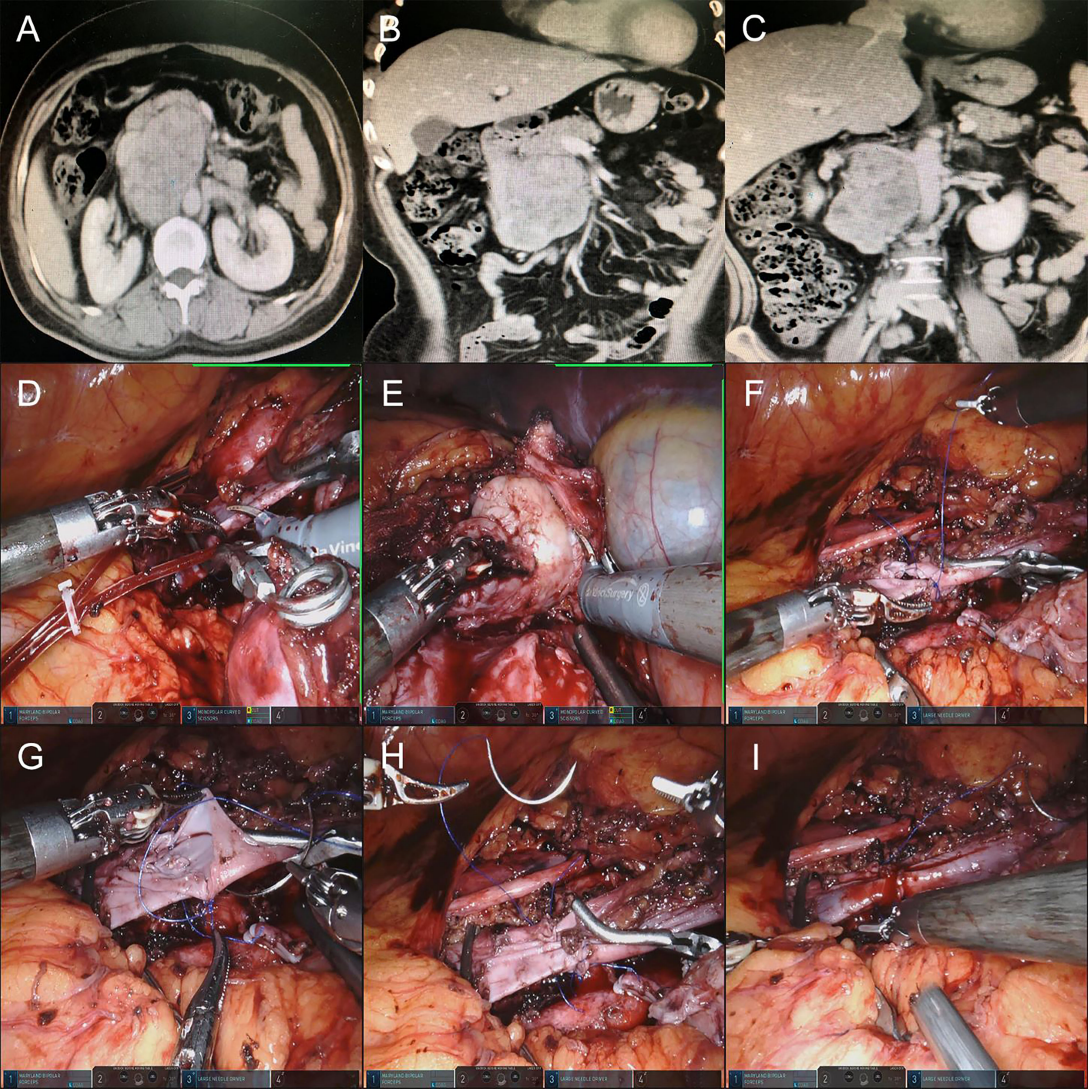
**(A–C)** Preoperative CTU images of the retroperitoneal tumor of patient 13. **(D)** Blocking the proximal and distal ends of the IVC by using a Bulldog clamp and cutting off the IVC. **(E)** Lift the proximal end of the IVC with separating forceps to free the tumor from the surface of the vessel. **(F–H)** After the tumor was removed, the broken end of the IVC was end-to-end anastomosed by a 3-0 vascular line. **(I)** The blockade of IVC and IVC blood flow patency recovery were resolved.

In this case, the patient had a 10-cm adrenal leiomyosarcoma just on the dorsal side of the IVC. The existence of IVC affected the surgeon to remove the tumor. Thus, the IVC was cut off after blocking the proximal and distal end of the IVC with a bulldog clamp. Lift the proximal end of the IVC with separating forceps to free the tumor from the surface of the vessel. After the tumor was removed, the broken end of the IVC was end-to-end anastomosed by 3-0 Prolene sutures for recovery of blood flow patency. The vascular blocking time was 107 min (**Figure 3**).

### Follow-Up Plan

Follow-up data were collected through outpatient departments and telephone interviews. The follow-up plan of patients was typically every 3 months with clinical examination, blood tests, chest and abdominal imaging (CT or MRI) in the first 2 years. Two years postoperatively, an individualized follow-up protocol according to the patient was established.

## Results

The baseline patients’ demographic details and symptoms are summarized in **Table 1**. The comparison between different surgical technique groups on parameters and outcomes are shown in **Table 2**. The median longest diameter of retroperitoneal tumors was 7.5 cm (range: 1.5-12 cm). RCC with IVCTT included 14 cases, with 3 cases of Mayo I, 9 cases of Mayo II and 2 cases of Mayo III. The presence of venous thrombus preoperatively occurred in 4 cases (patients 8, 11, 15, 17). Six patients (patients 3,7,9,11,12,15) were found distant metastasis preoperatively and recommended to use tyrosine kinase inhibitors, which needed to be used after surgery. Four patients (patients 8,11,15,17) were found venous thrombus through B-ultrasound preoperatively.

**Table 1:** Clinicopathological data of 17 patients with retroperitoneal tumors.

Patient No.	Age/ Gender	Symptoms	Tumor location	Tumor size (cm)	Mayo clinic classification (if applicable)	The presence of venous thrombus preoperatively	IVC management	Operation time(min)	Blood loss(ml)	Bood transfusion(ml)	Postoperative hospital stay (days)	Histology	Venous invasion in histology	Tumor grade	Severe complication	Distant metastasis	Neoadjuvant or adjuvant therapy	Follow-up
1	49/M	Pain	Right	5.5×3.5×3.5	2	No	①	246	200	0	9	Renal clear cell carcinoma	No	II	None	None	None	Alive and free of local recurrence in 17 months
2	57/F	Pain	Left	4.5×3.8×3	1	No	③	144	20	0	5	Renal clear cell carcinoma	No	I-II	None	None	Sunitinib (postoperatively)	Alive and free of local recurrence in 15 months
3	37/M	Pain	Right	6×5.5×4	1	No	③	245	2000	2000	9	Renal clear cell carcinoma	Lymphovascular invasion	II-III	None	Lung and bone (preoperatively)	Pazopanib (preoperatively and postoperatively) and radiotherapy (postoperatively)	Alive and free of local recurrence in 12 months; then loss to follow-up
4	62/F	Pain	Right	6×4.5×4.5	N/A	No	⑤	151	20	0	5	Leiomyosarcoma	No	High grade	None	None	None	Alive and free of local recurrence in 15 months
5	45/M	Hypertension	Right	4.7×5.4×7.4	N/A	No	②	283	3800	2000	16	Pheochromocytoma	Lymphovascular invasion	N/A	None	None	None	Alive and free of local recurrence in 15 months
6	46/F	Hematuria	Right	8×6.5×5	2	No	④	147	550	400	4	Type II papillary renal carcinoma	Venous wall invasion	III	None	None	None	Alive and free of local recurrence in 15 months
7	66/F	Pain, hematuria	Right	7.5×5.5×4.5	2	No	②	143	1000	400	6	Unclassified renal cell carcinoma	No	N/A	None	Lung (preoperatively)	Sunitinib (preoperatively and postoperatively)	Alive and free of local recurrence in 14 months
8	44/F	Fatigue	Right	9.5×7×7	2	Yes (left iliac vein, external iliac vein, femoral vein)	④	271	1000	800	8	Renal clear cell carcinoma	Lymphovascular invasion	III-IV	None	None	Sunitinib (postoperatively)	Alive and free of local recurrence in 12 months; then loss to follow-up
9	60/M	Pain	Right	3×2.7×2.5	1	No	①	125	50	0	4	Renal clear cell carcinoma	No	I-II	None	Left adrenal gland (preoperatively)	Pazopanib (preoperatively and postoperatively)	Alive and free of local recurrence in 13 months
10	61/M	Fatigue	Right	7.5×5.5	2	No	①	166	800	0	4	Renal clear cell carcinoma	No	III-IV	None	None	None	Alive and free of local recurrence in 13 months
11	72/M	Pain, hematuria	Right	8×4.6×4.5	2	Yes (distal end of IVC tumor thrombus)	④	138	500	400	9	urothelial carcinoma	Venous wall invasion	High grade	None	Lung and liver (postoperatively)	None	Alive and local recurrence in 8 months; then loss to follow-up
12	72/M	Asymptomatic	Left	7.5×3×4	2	No	①	263	200	0	7	Renal clear cell carcinoma	No	II/IV	None	Bone (preoperatively)	Pazopanib (preoperatively and postoperatively)	Alive and free of local recurrence in 12 months
13	47/F	Pain	Right	10.3×9.5×5.2	N/A	No	⑥	257	1550	400	6	Leiomyosarcoma	Lymphovascular invasion	High grade	None	None	None	Alive and free of local recurrence in 12 months
14	34/F	Asymptomatic	Right	1.5×1.2×1	3	No	①	130	300	400	6	Angioleiomyolipoma	No	N/A	None	None	None	Alive and free of local recurrence in 12 months
15	39/M	Hematuria	Right	7×6×5.5	2	Yes (bilateral iliac veins)	④	399	1300	400	9	Type II papillary renal carcinoma	Venous wall invasion	III	None	Lung (preoperatively)	Pazopanib (preoperatively and postoperatively)	Alive and free of local recurrence in 12 months
16	33/M	Pain	Right	12×8×5	3	No	②	148	300	0	6	Angioleiomyolipoma	No	N/A	None	None	None	Alive and free of local recurrence in 10 months
17	50/F	Pain	Right	9×6.5×3.5	2	Yes (distal end of IVC tumor thrombus)	④	148	200	400	5	Renal clear cell carcinoma	No	II-III	None	None	None	Alive and free of local recurrence in 9 months

① Conventional total occlusion of IVC and its branches; ②Delayed occlusion of proximal IVC technique; ③ IVC resection by Satinsky clamp; ④IVC ligation; ⑤ IVC reconstruction by bovine pericardial grafts; ⑥ IVC segmental resection and reconstruction.N/A, Not Applicable.

**Table 2:** Comparison between different surgical technique groups on parameters and outcomes.

Surgical technique	Patient No.	n	Tumor size/median (range) (cm)	Operation time/median (range) (min)	Blood loss/median (range) (ml)	Bood transfusion/median (range) (ml)	Postoperative hospital stay/median (range) (days)	Follow-up
Conventional total occlusion of IVC and its branches	patient 1,9,10,12,14	5	5.5 (1.5-7.5)	166 (125-263)	200 (50-800)	0 (0-400)	6 (4-9)	No local recurrence
Delayed occlusion of proximal IVC technique	patient 5,7,16	3	7.5 (7.4-12)	148 (143-283)	1000 (300-3800)	400 (0-2000)	6 (6-16)	No local recurrence
IVC resection by Satinsky clamp	patient 2,3	2	5.25 (4.5-6)	194.5 (144-245)	1010 (20-2000)	1000 (0-2000)	7 (5-9)	No local recurrence
IVC ligation	patient 6,8,11,15,17	5	8 (7-9.5)	148 (138-399)	550 (200-1300)	400 (400-800)	8 (4-9)	No local recurrence
IVC reconstruction by bovine pericardial grafts	patient 4	1	6	151	20	0	5	No local recurrence
IVC segmental resection and reconstruction	patient 13	1	10.3	257	1550	400	6	No local recurrence
Total	/	17	7.5 (1.5-12)	151 (125-399)	500 (20-3800)	400 (0-2000)	6 (4-16)	No local recurrence

In terms of IVC management, 5 patients (patients 1, 9, 10, 12, 14) had conventional total occlusion of the IVC and its branches, 3 patients (patients 5, 7, 16) underwent the DOPI technique, 2 patients (patients 2, 3) underwent IVC resection by Satinsky clamp, 5 patients (patients 6, 8, 11, 15, 17) underwent IVC ligation and 1 patient (patient 4) underwent IVC reconstruction by bovine pericardial grafts. In one case (patient 6), it was difficult to suture or reconstruct the invaded IVC because of the tumor invasion of the IVC wall. Therefore, we performed complete tumor resection and IVC ligation as mentioned above. The retroperitoneal tumor of patient 13 was located on the dorsal side of the IVC, and the IVC just crossed the tumor. We performed IVC transection for more convenient removal of the tumor and then anastomosed the IVC with a 3-0 Prolene suture *via* a robotic-assisted laparoscopic approach. In another case (patient 5), IVC was injured by mistake during the operation, and it was difficult to stop bleeding with electrocoagulation. We sutured and repaired IVC rip slits with a 4-0 Prolene vascular line.

The median operation time was 151 min (range: 125-399 min), and blood loss was 500 ml (range: 20-3800 ml). The average operation time was 200 ± 74.9 min and blood loss was 811 ± 934.4ml. Ten patients (patients 3,5,6,7,8,11,13,14,15,17) were found anemia and required blood transfusion. After the operation, only patient 5 was treated with low molecular weight heparin because he had venous thrombus but did not undergo IVC ligation. There was no severe complication perioperatively.

Of the 17 patients, 8 patients had renal clear cell carcinoma, 2 patients had type II papillary renal carcinoma, 2 patients had leiomyosarcoma, 1 patient had urothelial carcinoma, 2 patients had angioleiomyolipoma, 1 patient had pheochromocytoma, and 1 patient had unclassified renal cell carcinoma. The surgical margin of all the patients were negative in histology. In addition to the patients (patient 3,7,9,12,15) who found metastasis preoperatively and continued to use TKIs after operation, patient 2 and patient 8 were also recommended to use Sunitinib because of lymph node metastasis.

The follow-up time of 17 patients was 8 to 17 months (median: 12 months). During follow-up, there was no new venous thrombotic events. Patient 11 with urothelial carcinoma was found lung and liver metastasis in 8 months after surgery and did not receive any adjuvant treatment because of poor health status. No local recurrence or overall death was found during follow-up.

## Discussion

Retroperitoneal tumors may be closely related to the IVC due to their large volume or direct invasion of the IVC. Resection of the IVC is required in 15–25% of patients undergoing nephrectomy for RCC with TT (10). Since the anatomic relationship between retroperitoneal tumors and IVCs varies, we performed different surgical strategies. In this study, we divided these surgical procedures into 4 categories in detail. The purpose of our research was to completely remove the tumor *via* a robot-assisted laparoscopic approach. In total, there were no major complications perioperatively, as well as recurrence and death during follow-up.

It is difficult to evaluate the relationship between retroperitoneal tumors and IVC preoperatively. Sohaib et al. (11) revealed that the sensitivity of MRI to invasion of the IVC wall is 92%. Zini et al. (12) reviewed a 32 case series with IVC tumor thrombectomy and concluded that an anteroposterior diameter of IVC > 18 mm or a diameter of the entrance of the renal vein into the IVC > 14 mm on preoperative MRI was a risk factor for invasion of the IVC wall. The sensitivity of this criterion reached approximately 90%. Scott et al. (13) explored several radiographic and clinical factors to create a model for patients who needed to undergo IVC resection or reconstruction (ROR). IVC involvement was the strongest predictor of vascular ROR. If IVC is invaded at least 135° circumference, nearly 50% of patients require ROR. However, the final surgical strategy should be determined according to the specific findings during the operation. Obviously, there is no need to perform ROR of IVC when the tumor lacks contact with it.

The surgical technique of conventional total occlusion of the IVC and its branches seems to be the most common method to handle the IVC, regardless of whether resection of the IVC wall is necessary. Based on this technique, some other modified methods have been derived, such as the DOPI technique, IVC resection by Satinsky clamp, and IVC ligation. The DOPI technique applies the method of increasing pneumoperitoneum pressure instead of blocking to reduce blood reflux on the proximal end of the IVC. Therefore, this is only suitable for patients whose IVC wall is not invaded by the tumor (9). Before surgery, it is necessary to use CT or MRI to confirm the extent and invasion of TT. The IVC and its branches should be fully exposed, and the vascular blocking band needs to be made for both free and blocking intraoperatively. In our previous single-situation research, we performed 17 patients who underwent retroperitoneal laparoscopic radical nephrectomy and thrombectomy with the DOPI technique. Moreover, we went a step further to put this technique in a robot-assisted laparoscopic approach and achieved the same results.

After partial resection, IVC can be reconstructed with primary closure or patch repair. Satinsky clamp is routinely used in primary closure with 4-0 Prolene sutures. The use of the Satinsky clamp could avoid full IVC occlusion and does not affect the blood reflux of the unviolated renal vein, the distal end of the IVC or its branches. However, the dissociation and vascular blocking band of the IVC is still essential for unexpected emergencies. The indication of this technique is RCC with Mayo level 0 or 1 TT, which exists in the renal vein or just enters the IVC. The size of the defect and tension should be considered in the primary repair and reconstruction of IVCs. The primary repair, including using a Satinsky clamp, can be done by longitudinally cutting and transverse seams when tension permits. The extremely narrow IVC after suture may cause thrombosis or affect the reflux of the distal vena cava. The use of patch grafting could settle the case, and various autologous, biologic, and synthetic materials are used to avoid these complications (14). Patch repair mainly consists of polytetrafluoroethylene (PTFE) and bovine pericardium, which are typically necessary when >50% of the circumference of the IVC is removed (12). Illuminati et al. (15) summarized the PTFE graft replacement of IVC tumor at different locations in 10 caval leiomyosarcoma patients. PTFE graft replacement of IVC could obtain the preservation of venous return increasingly. In the absence of oral anticoagulants, a satisfactory patency rate of 67% in 5 years can still be obtained. The advantage of using PTFE as a caval substitute is practical to patch the missing part of IVC without narrowing it; PTFE has various sizes, which significantly reduces the possibility of anastomotic mismatch; PTFE is more tough to resist abdominal visceral compression compared with biological patch (16). In our study, we used bovine pericardium for patch repair of defective IVCs. The area of bovine pericardium should be slightly smaller than the defect, and the smooth surface must be the intimal surface of blood vessels to prevent thrombosis. Regardless of how the IVC is repaired or anastomosed, the vascular intima should be tightly aligned, which conforms to normal physiological structure.

If lower extremity venous thrombosis is found in patients before operation, we suggest to add therapeutic dose of short-term anticoagulant therapy before operation, such as low molecular weight heparin, which should be stopped half a day before operation to reduce intraoperative bleeding. The role of anticoagulant therapy is to prevent new thrombosis and thrombus shedding leading to pulmonary embolism. Moreover, IVC ligation is more recommended for patients requiring IVC management, which could block the access of the distal venous thrombus of IVCTT into the pulmonary circulation. Some researchers advocate for the use of routine systemic anticoagulation after IVC reconstruction to prevent VTE and graft thrombosis events (17, 18). In contrast, an experience of 65 patients who underwent IVC reconstruction from Caitlin showed that the results did not support postoperative routine anticoagulation because the incidence of VTE after IVC reconstruction was approximately 22% with minimal severe sequelae of this complication (4). However, they also emphasized that it certainly deserved to be monitored carefully with self-reported symptoms and lower extremity duplex ultrasound. In our study, 4 patients were found venous thrombus by B-ultrasound preoperatively and received subcutaneous injection of low molecular weight heparin. IVC ligation was performed in these cases and anticoagulant therapy was not continued postoperatively. For other cases, we did not perform postoperative systemic anticoagulation routinely with consideration of the relatively low incidence of VTE severe complications reported and increased potential risks of bleeding. During follow-up, there was no evidence of venous thrombosis. Only patient 5 had an IVC thrombus without IVC ligation postoperatively and was treated with low molecular weight heparin. This patient was followed up for 15 months without progression of thrombosis and tumor recurrence.

IVC ligation is suitable for patients with extensive invasion of the IVC wall (mostly >50% circumference of the IVC) or thrombus at the distal end of IVCTT. IVC can be evaluated preoperatively or after incision of IVC during operation. IVC ligation is recommended for patients with right RCC with IVCTT, which does not affect left renal function in most cases. A few patients need dialysis treatment for 2 ~ 4 weeks, and residual renal function can be recovered due to the reconstruction of collateral circulation of the left renal vein, which consists of the gonadal vein, adrenal vein, lumbar vein, etc. (19). For the left tumor, ligation could be tolerated only if collateral circulation of the IVC is preserved (17). It deals with IVCTT first in the surgical procedure of the robot-assisted laparoscopic approach. After IVC ligation, the proximal end of the IVC can be lifted from bottom to top, which contributes to the dissociation of the IVCTT.

In recent years, whether reconstruction is needed after IVC ligation has been a focus question. An increasing number of researchers have suggested that IVC reconstruction after resection is only necessary in patients who have poor preoperative development of collateral circulation or who have unstable hemodynamics intraoperatively (14) (20). Extremity edema and postoperative renal dysfunction are common complications after IVC ligation. The incidence of these complications is approximately 8.3% (21). If patients suffer from severe venous sequelae or postoperative compartment syndrome after IVC ligation, IVC reconstruction can be performed, and the best time is within one year after the primary operation (22). Brian (20) suggested that IVC resection without reconstruction was well tolerated in patients with large retroperitoneal masses, which constantly indicated well-established collaterals preoperatively due to severe IVC obstruction. Our experience is consistent with previous literature. The collateral circulation can mainly provide sufficient venous reflux, especially in cases of recovery of lower limb edema, suggesting good compensation. When technical demanding, conversion to open surgery was usually a good option. In addition, if there is venous thrombus at the distal end of the tumor thrombus in the IVC lumen, the procedure of vessel reconstruction should not be applicable to avoid pulmonary embolism caused by thrombus fall-off.

IVC transection and anastomosis for more convenient removal of tumors is a specific and rare surgical technique. The location of the tumor was hidden on the dorsal side of the IVC, which increased the difficulty of separation. The key point of the operation lies in the presence of lumbar vein branches of the IVC and neovascularization of the tumor, which may lead to increased intraoperative bleeding and operation time. The anastomosis of the IVC usually completes the suture of the posterior wall first and then the anterior wall. At present, this surgical technique has been feasible with experienced surgeons in robot-assisted laparoscopic approaches, and the operation time can be controlled within a certain range. Moreover, the isolation of IVCs does not increase the risk of massive bleeding because the IVC has been blocked.

Our study also has some limitations. First, this is a retrospective study with a short follow-up time. Three patients were lost to follow-up and the latest survival and recurrence were not available. Due to the wide variety of surgical strategies for IVC, the number of cases in each category is minimal. Second, the decision to perform different surgical strategies might be partly subjective and affected by surgeon experience according to tumor circumstance intraoperatively. Finally, IVC artificial vessel reconstruction is not included in our technical category. There are fewer cases because of the presence of complications such as thrombosis and infection. Moreover, this technique is hard to perform completely by robot-assisted laparoscopic systems.

## Conclusion

We performed several surgical strategies for retroperitoneal tumors with IVC-invaded patients and considered that it was safe and feasible in experienced centers. In robot-assisted laparoscopic approaches, these surgical techniques are helpful to completely remove the tumor for better oncological prognosis and restore the blood reflux of the IVC as much as possible to ensure fewer postoperative complications.

## Data Availability Statement

The raw data supporting the conclusions of this article will be made available by the authors, without undue reservation.

## Ethics Statement

The studies involving human participants were reviewed and approved by the Peking University Third Hospital ethics committee. The study was approved by the institutional review board of the hospital involved (the number of ethics approval: No. 2018-396-01). The patients/participants provided their written informed consent to participate in this study. Written informed consent was obtained from the individual(s) for the publication of any potentially identifiable images or data included in this article.

## Author Contributions

LL and ST: Study design, Data collection, Data analysis, Writing, Revision. ZL, CL, HZ, XT, GW, SZ: Study design, Data collection. LM: Study design, Data collection, Revision. All authors contributed to the article and approved the submitted version.

## Funding

This study was supported by the National Nature Science Foundation of China (NO. 81771842) and the National Nature Science Foundation of China (NO. 82072211).

## Conflict of Interest

The authors declare that the research was conducted in the absence of any commercial or financial relationships that could be construed as a potential conflict of interest.

## Publisher’s Note

All claims expressed in this article are solely those of the authors and do not necessarily represent those of their affiliated organizations, or those of the publisher, the editors and the reviewers. Any product that may be evaluated in this article, or claim that may be made by its manufacturer, is not guaranteed or endorsed by the publisher.

## Abbreviations

IVC, inferior vena cava; RCC, renal cell carcinoma; TT, tumor thrombus; VTE, venous thromboembolic; TKI, tyrosine kinase inhibitor; CTU, computed tomography urography; MRI, magnetic resonance imaging; DOPI, delayed occlusion of proximal IVC; PTFE, polytetrafluoroethylene.
